# Suppression of Oxygen Vacancy Defects in sALD-ZnO Films Annealed in Different Conditions

**DOI:** 10.3390/ma13183910

**Published:** 2020-09-04

**Authors:** Ming-Jie Zhao, Zhi-Tao Sun, Zhi-Xuan Zhang, Xin-Peng Geng, Wan-Yu Wu, Shui-Yang Lien, Wen-Zhang Zhu

**Affiliations:** 1 School of Opto-electronic and Communication Engineering, Xiamen University of Technology, Xiamen 361024, China; 2015000077@xmut.edu.cn (M.-J.Z.); 18359225079@163.com (Z.-T.S.); 1922031023@stu.xmut.edu.cn (Z.-X.Z.); gexipe@163.com (X.-P.G.); wzzhu@xmut.edu.cn (W.-Z.Z.); 2 Fujian Key Laboratory of Optoelectronic Technology and Devices, Xiamen University of Technology, Xiamen 361024, China; 3 Department of Materials Science and Engineering, Da-Yeh University, Changhua 51591, Taiwan; wywu@mail.dyu.edu.tw

**Keywords:** zinc oxide, oxygen vacancy defects, spatial atomic layer deposition, annealing, crystallinity

## Abstract

Zinc oxide (ZnO) has drawn much attention due to its excellent optical and electrical properties. In this study, ZnO film was prepared by a high-deposition-rate spatial atomic layer deposition (ALD) and subjected to a post-annealing process to suppress the intrinsic defects and improve the crystallinity and film properties. The results show that the film thickness increases with annealing temperature owing to the increment of oxide layer caused by the suppression of oxygen vacancy defects as indicated by the X-ray diffraction (XRD) and X-ray photoelectron spectroscopy (XPS) spectra. The film transmittance is seldom influenced by annealing. The refractive index increases with annealing temperature at 300–700 °C, possibly due to higher density and crystallinity of the film. The band gap decreases after annealing, which should be ascribed to the decrease in carrier concentration according to Burstein–Moss model. The carrier concentration decreases with increasing annealing temperature at 300–700 °C since the oxygen vacancy defects are suppressed, then it increases at 800 °C possibly due to the out-diffusion of oxygen atoms from the film. Meanwhile, the carrier mobility increases with temperature due to higher crystallinity and larger crystallite size. The film resistivity increases at 300–700 °C then decreases at 800 °C, which should be ascribed primarily to the variation of carrier concentration.

## 1. Introduction

Zinc oxide (ZnO) is a popular material which has been paid much attention due to its excellent optical and electrical properties. The applications of ZnO include solar cells, light-emitting diodes (LEDs), gas sensors, and thin-film transistors (TFTs) [1,2,3,4,5]. ZnO film can be prepared by several techniques, such as pulsed laser deposition (PLD), sputtering, chemical vapor deposition (CVD), solution-based methods, atomic layer deposition (ALD), and molecular beam epitaxy (MBE) [6,7,8,9,10,11]. Among these techniques, ALD is distinguished for the self-limiting surface reaction between the precursors which is confined to the substrate surface. Therefore, it is possible to control the thin film growth at the atomic layer scale and hence high-quality thin film with precise control of film growth can be obtained by this technique. However, the deposition rate of conventional thermal ALD is somewhat low due to the long purge time needed to remove the residues of the reaction, which limits its application in industrial manufacturing. Recently, the disadvantage of thermal ALD has been avoided by the development of spatial ALD (sALD), where the precursors are dosed to separated zones of the reactor divided by a nitrogen gas curtain. When the substrate moves horizontally under the precursor spray heads, it was exposed to the precursors successively and the reaction occurs on its surface. In this way, the purge step can be omitted [12,13]. Therefore, the atmospheric sALD process is more cost-efficient than other vacuum-based process. Therefore, sALD is emerging as a promising technique for mass manufacture owing to the possible high throughput.

Generally, the properties of ZnO film strongly depend on the intrinsic defects. The defective structure of ZnO has received much attention for various optoelectronic and catalytic devices. However, the oxygen defects in ZnO film can be favorable or harmful depending on their states and applications. As one of the major defects, the oxygen vacancy defects in oxide film may act as either donor or acceptor in the band gap depending on their coordinating conditions and charged states [14,15,16,17]. On one hand, oxygen vacancy defects with high coordination or that are negatively charged serve as shallow donors that donate free carriers to the conduction band and contribute to the conduction of the film, which are favorable for some applications such as transparent conductive oxide film. However, oxygen vacancy defects need to be carefully controlled in some applications such as TFTs, where the free carrier concentration should be controlled at a low level so that they can be depleted to achieve the off-state of the TFTs [18,19,20]. On the other hand, oxygen vacancy defects with low coordination, that are neutrally or positively charged, serve as deep trap states in the band gap. They may deteriorate or cause instability of the film properties. For instance, they may deteriorate the subthreshold characteristics and induce instability of the oxide TFTs [21]. This kind of oxygen vacancy defects should be suppressed. Annealing treatment after film deposition has been reported to be an effective way to reduce the defects and improve the crystallinity [22,23,24,25,26,27]. There have been some reports regarding the post-annealing effects on ZnO film grown by conventional thermal ALD and other techniques [18,28,29,30,31,32]. However, the annealing effects on sALD-ZnO film are seldom reported. In fact, the post-annealing conditions are crucial for improving the film properties. In this study, ZnO films with thickness of 100 nm were prepared by a high-deposition-rate sALD process, which has similar gain per cycle (GPC) values (~0.95 Å/cycle) to other ALD processes [33,34]. However, it costs only 4 s for one sALD cycle, and tens of seconds for one plasma-enhanced or thermal ALD cycle [35,36]. As a result, it spends about 70 min to obtain an 100-nm-thick ZnO film by an sALD process, whereas it spends at least several times longer to obtain similar film thickness by other ALD techniques. Therefore, the sALD process is more promising for industrial applications. After film deposition, the films were subjected to a post-annealing process. It was found that the oxygen vacancy defects were suppressed by the annealing process. The effects on the film properties were also analyzed. An effective way to suppress the oxygen vacancy defects is provided in this work and might be useful for some applications.

## 2. Materials and Methods

ZnO films were grown on quartz substrate, or silicon wafer by a spatial ALD facility using diethylzinc (DEZ) and deionized water (H_2_O) vapor as precursors at a substrate temperature of 90 °C. The substrate was placed beneath the spray heads at a distance of 0.3 mm and moved back and forth at a speed of 15 cm/s. The parameters for the film deposition and annealing process are summarized in Table 1. Other detailed descriptions of the film deposition process have been reported elsewhere [37]. The ZnO films were annealed at 300–800 °C in an air ambient for 1 h at each temperature separately by a thermal annealing process (TAP) after deposition.

The film thickness (*t*) was measured by an ellipsometer (M-2000, J. A. Woollam Co., Inc., Lincoln, NE, USA). The refractive index (*n*) of the ZnO film grown on silicon wafer was obtained by a spectroscopic ellipsometer in the wavelength range of 350–800 nm. The *n* value of ZnO film was evaluated by an air roughness model, which was built up by a four-layer structure consist of “air, air/ZnO, ZnO, silicon substrate”, where the ZnO layer was fitted by a Tauc-Lorentz model. The ZnO films were grown on quartz substrate for the measurements of the transmittance (*T*) and reflectance (*R*) spectra using a spectrometer (MFS-630, Hong-Ming Technology, New Taipei City, Taiwan) in the wavelength of 380–1000 nm taking the air as the background. The light was incident from the air to the film. The crystal structure of the ZnO films was characterized by grazing-incidence X-ray diffraction (GIXRD, Rigaku TTRAXⅢ, Ibaraki, Japan) spectra with an X-ray wavelength of 0.154 nm and a minimum scanning step of 0.02°. The chemical states of the elements were analyzed by an X-ray photoelectron spectroscope (XPS, ESCALAB 250Xi, Thermo Fisher, Waltham, MA, USA) equipped with an Al anode (Al-Kα) in a wide range of binding energy. The morphology of the film surface was observed by a field-emission scanning electron microscope (FESEM sigma 500, Zeiss, Oberkochen, Germany). The electrical properties of the films were detected by a Hall effect measurement system (HMS5000, Side Semiconductor Technology, Shanghai, China).

## 3. Results and Discussion

**Figure 1:**
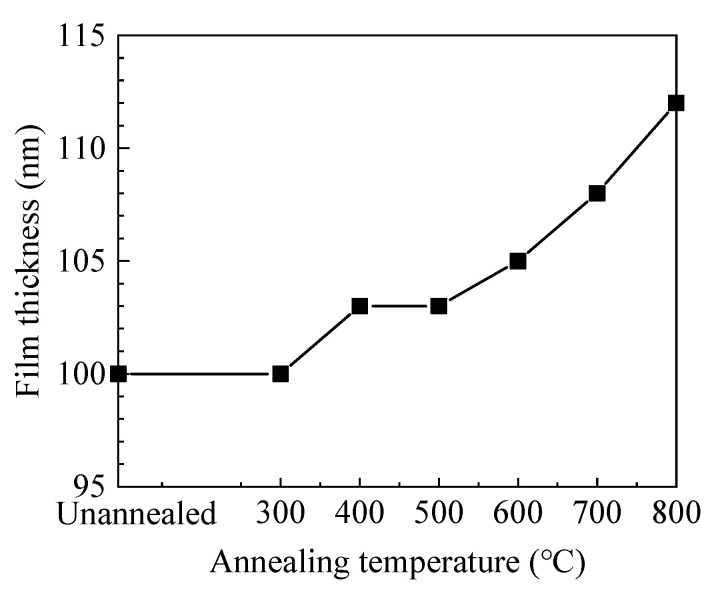
The variation of film thickness with annealing temperature.

As shown in Figure 1, the film thickness increases with annealing temperature. Usually, annealing treatment would cause structural relaxation and densification of the film, resulting in the decrease of film thickness. In this case, the increase of film thickness should be ascribed to the increment of oxide layer and will be further discussed in the XPS results.

**Figure 2:**
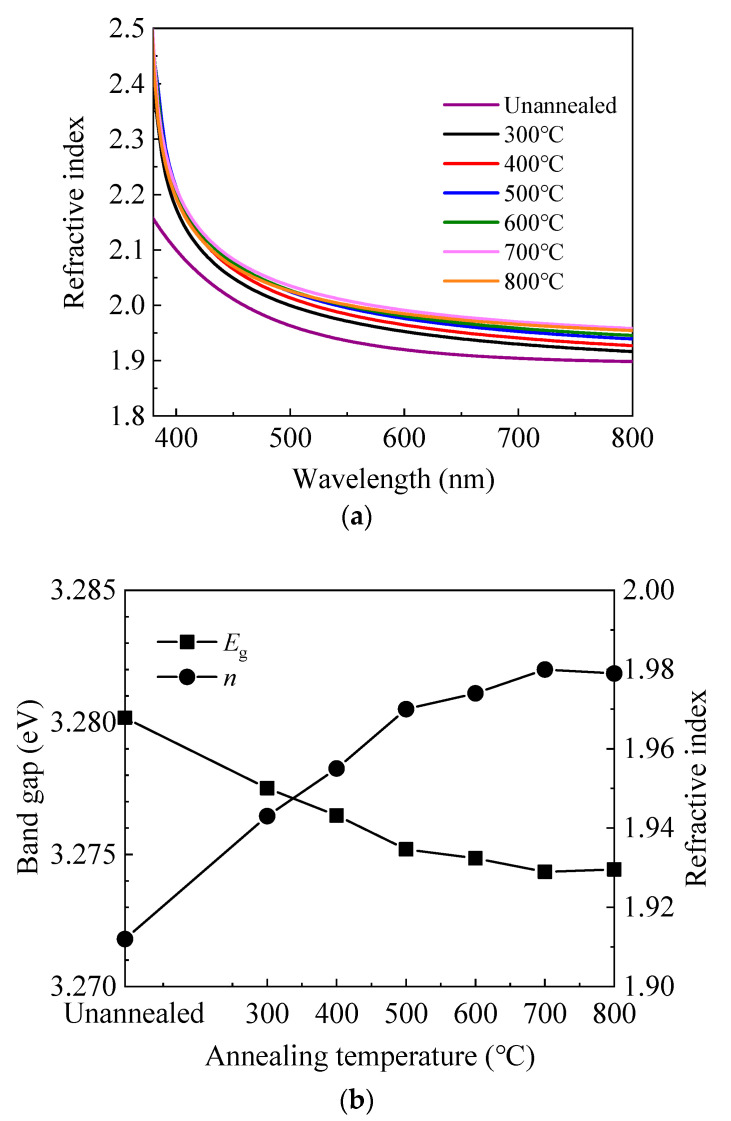
(**a**) The wavelength-dependent refractive index (*n*) and (**b**) the variation of refractive index at 630 nm and band gap calculated from the refractive index with annealing temperature.

Figure 2a shows the wavelength-dependent refractive index (*n*) of sALD-ZnO films on silicon wafer. In some applications such as solar cells, the refractive index at 630 nm is often concerned, so it is shown as a function of annealing temperature. The value of ≈1.95 at 630 nm is in accordance with those reported in the literature [12,34,38]. In addition, the refractive index continuously increases with annealing temperature at 300–700 °C, then slightly decreases at 800 °C. The variation of refractive index can be a reflection of the change in the film density since they are closely related [38,39]. Namely, the film density increases with annealing temperature at 300–700 °C, then slightly decreases at 800 °C. It is possible that annealing at 300–700 °C induces structural relaxation and densification of the film, and hence increasing the refractive index. Another possible reason for the increase of refractive index can be the enhancement in crystallinity as indicated by the XRD and scanning electron microscopy (SEM) results. However, annealing at 800 °C might cause out-diffusion of zinc or oxygen atoms, leading to the decrease of refractive index [40,41,42]. The optical band gap (*E*_g_) was also calculated from the refractive index at 630 nm according to the following model [43]:

*n*^2^ = 1 + [13.6/(*E*_g_ + 3.4)]^2^ (1)

As shown in Figure 2b, the optical band gap decreases with increasing annealing temperature at 300–700 °C, then slightly increases at 800 °C. The decrease of optical band gap can be ascribed to the non-degeneracy of the energy states caused by the decrease of carrier concentration according to the Burstein–Moss model [12,44]. Similarly, the slight increase of band gap at 800 °C should be ascribed to the increase of carrier concentration.

**Figure 3:**
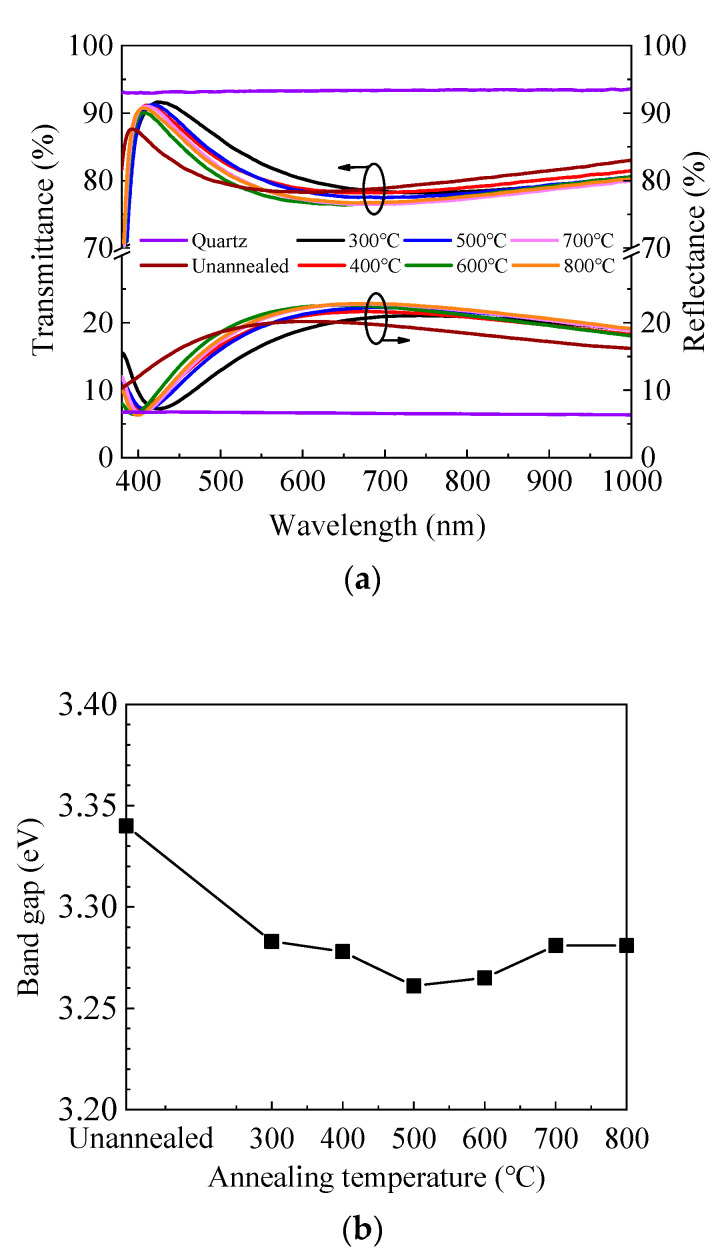

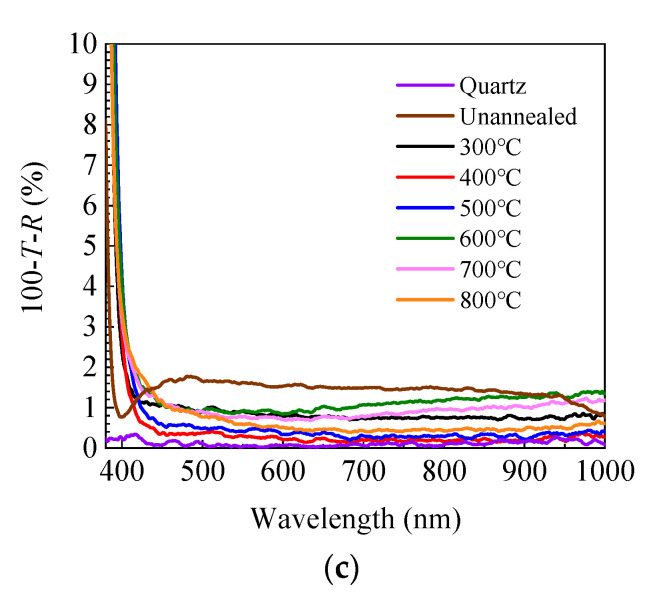
(**a**) Transmittance and reflectance spectra of sALD-ZnO films annealed at different temperatures; (**b**) dependence of optical band gap (*E*_g_) of sALD ZnO films on annealing temperature; (**c**) optical loss spectra of sALD-ZnO films annealed at different temperatures.

Figure 3a shows the transmittance and reflectance spectra of ZnO/quartz samples annealed at different temperatures. The transmittance is high in the visible and near-infrared light range for all samples. It can be seen that the transmittance spectra change slightly with annealing temperature and exhibit an inverse correlation with the reflectance spectra. Therefore, the variation of transmittance spectra should be attributed primarily to the different reflection conditions caused by the different refractive indexes and film thicknesses of ZnO films annealed in different conditions. In addition, the transmittance dramatically decreases at the short wavelength around 400 nm, which corresponds to the absorption caused by the band-to-band transition. The absorption edge shifts to the longer wavelength region after annealing, indicating the decrease of optical band gap (*E*_g_). To verify this, the optical band gap was also extracted by Tauc’s plot as expressed by the equation [45]:

(αhν)^2^ = A (hν − *E*_g_) (2)

where α is the absorption coefficient, hν is the energy of the incident light, and A is a constant factor. Figure 3b shows the dependence of the optical band gap on annealing temperature. The optical band gap and its developing trend are broadly in line with that calculated from the refractive index. The small fluctuation of the band gap value is probably due to the fitting error of the Tauc plots. The optical loss spectra have been calculated as expressed by 100-*T*-*R* to reflect the absorption and scattering of the incident light and plotted in Figure 3c. The optical loss is less than 2% in the range of 450–1000 nm for the unannealed sample and further decreases to less than 1% after annealing. Therefore, the optical loss does not have a significant effect on the transmittance spectra in the range of 450–1000 nm. However, it dramatically increases at the shorter wavelength (380–450 nm) due to the absorption by band-to-band transition, leading to the dramatic decrease of transmittance.

**Figure 4:**
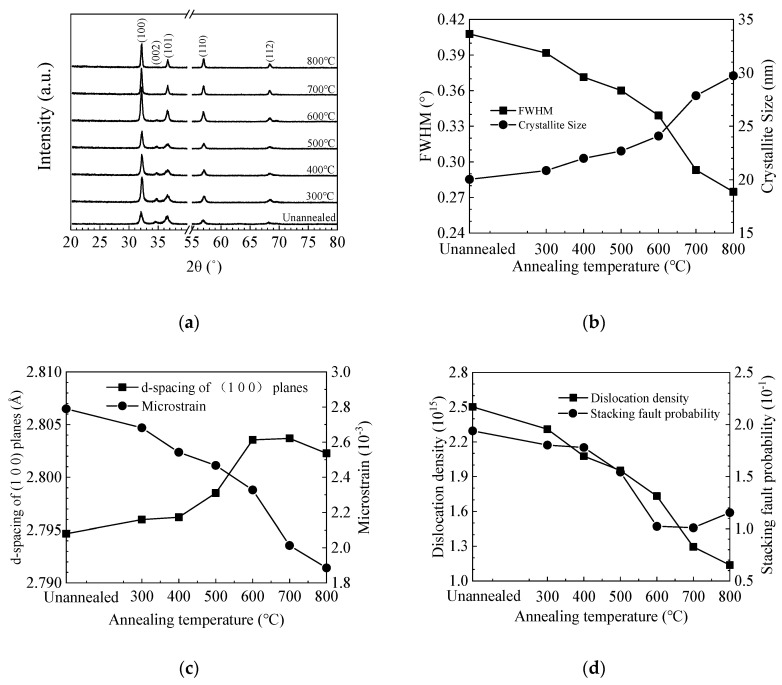
(**a**) Grazing-incidence X-ray diffraction (GIXRD) spectra of sALD-ZnO films annealed at different temperatures; (**b**) variation of full width at half maximum (FWHM) for the (1 0 0) peak and the crystallite size of sALD ZnO films with annealing temperature; dependence of (**c**) d-spacing of (1 0 0) planes, microstrain, (**d**) dislocation density and stacking fault probability on the annealing temperature.

Figure 4a shows the GIXRD spectra of ZnO films deposited on silicon wafer. The diffraction peaks can be well identified to the (100), (002), (101), and (110) planes of the hexagonal wurtzite ZnO lattice, among which the (100) peak has the strongest intensity [46,47]. Moreover, a weak (002) peak is also observed for the unannealed and low-temperature (300–500 °C) annealed films, then disappears at higher annealing temperature. This might be responsible for the increase of film resistivity as shown in the Hall effect results since the (002) plane is the most compact plane in the wurtzite ZnO lattice [48]. The intensity of other peaks increases with annealing temperature, indicating the increase of crystallinity. It is worth noting that the diffraction peaks shift with annealing temperature, which suggests a lattice expansion or contraction. Various micro-structural parameters such as crystallite size (*D*), interplanar distance (d-spacing), microstrain (*ε*), dislocation density (*δ*), and stacking fault probability (*α*) were also calculated from the XRD data and plotted in Figure 4b–d [49,50]. The average size of the (100)-orientated crystallites was estimated using Scherrer’s equation [51]:

*D* = *kλ*/(*β*cos*θ*) (3)

where *k* is a shape factor, *λ* is the wavelength of the X-ray, *β* and *θ* are the full width at half maximum (FWHM) and the Bragg angle of the (1 0 0) peaks, respectively. The interplanar distance of the (1 0 0) planes was calculated from the Bragg formula [52]:

2*d*sin*θ* = *nλ* (4)

where *d* is the interplanar distance, *n* is the order of diffraction. The microstrain *ε* was calculated as:

*ε* = *β*cot*θ* − *λ*/(*D*sin*θ*) (5)

where *D* is the crystallite size of the preferentially (100)-orientated crystallites. The dislocation density *δ* was calculated as:

*δ* = *n*/*D*^2^ (6)

where *n* is a factor. The stacking fault probability (*α*) was calculated as:

(7) $$\alpha =\left(2\pi^{2}/45\sqrt{3}\right)\left[\Delta \left(2\theta \right)/\tan\theta \right]$$

where Δ(2*θ*) is the peak shift. Figure 4b shows the variation of FWHM value and crystallite size with annealing temperature. The FWHM value decreases with increasing temperature. Accordingly, the crystallite size increases with increasing temperature. In addition, the increase of crystallite size is more significant at 700–800 °C. The interplanar distance for the unannealed ZnO film is ~2.795 Å, which is smaller than the standard value of 2.816 Å, suggesting a lattice contraction of the film, possibly due to the existence of a large number of oxygen vacancy defects in the lattice [33,53]. The interplanar distance increases with annealing temperature at 300–700 °C, implying that the vacancy defects are repaired, possibly by absorbing oxygen atoms from the ambient. However, the interplanar distance slightly reduces when the annealing temperature further increases to 800 °C, possibly due to the creation of new vacancy defects. Zn out-diffusion at similar annealing temperature (900 °C) has been observed by other group [40,41,42]. Oxygen out-diffusion may be another possible reason for the decrease of interplanar distance as inferred from the XPS results. The development of interplanar distance is consistent with the variation of refractive index since the film density may be influenced by the vacancy defects. Accordingly, the microstrain releases as the annealing temperature increases mainly due to the restoration of the lattice contraction indicated by the shift of diffraction peaks to the standard position and the increase of interplanar distance approaches the standard value. The increase of crystallite size also contributes to the release of microstrain owing to the decrease of *β* value. Therefore, the microstrain keeps decreasing at 800 °C regardless of the decrease of interplanar distance. The dislocation density decreases with increasing annealing temperature owing to the increase of crystallite size. The stacking fault probability decreases with increasing annealing temperature at 300–700 °C due to the reduction of the peak shift as well as the variance of interplanar distance compared with the standard value. Then, it slightly increases at 800 °C due to the out-diffusion of oxygen atoms.

**Figure 5:**
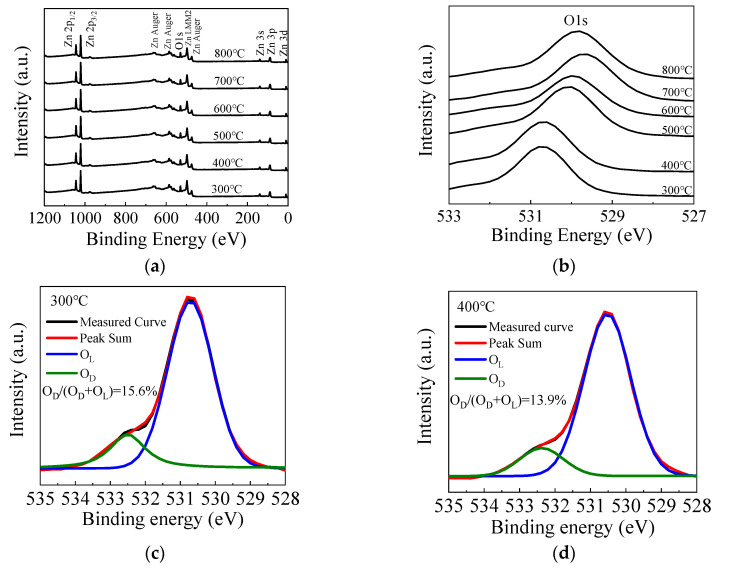

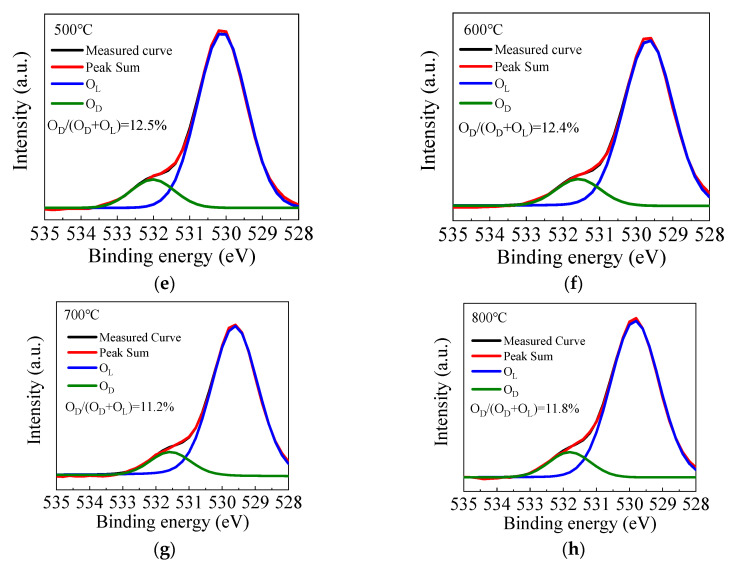
(**a**) The fully scanned X-ray photoelectron spectroscopy (XPS) spectra and (**b**) the O 1s peak of the sALD-ZnO films annealed at 300–800 °C; (**c**–**h**) the high-resolution spectra of O 1s peak of the sALD ZnO films annealed at 300–800 °C.

Figure 5a shows the fully scanned XPS spectra of the ZnO films with different annealing temperatures. The observed peaks can be assigned to Zn or O element [54]. Figure 5b shows the O 1s peak of the ZnO films annealed by different temperatures. The O 1s peak shifts towards the lower binding energy direction as the annealing temperature increases from 300 to 700 °C, due to the filling of oxygen vacancies by absorbing oxygen from the ambient, leading to higher valence electron density around the oxygen and hence a stronger screening effect of the binding energy. However, the peak slightly shifts back to the higher binding energy direction when the annealing temperature further increases to 800 °C, suggesting more oxygen vacancy defects, possibly due to the out-diffusion of oxygen atoms from ZnO film at such a high annealing temperature. Figure 5c–h shows the high-resolution spectra of O 1s peaks for ZnO films annealed at 300–800 °C. The O 1s peak can be fitted by two constituent peaks. The peaks located at around 529.8 eV (O_L_) and 531.4 eV (O_D_) are related to oxygen in a stoichiometric hexagonal wurtzite ZnO lattice and that with oxygen vacancy defects, respectively [55,56]. In the literature, a peak around 534.3 eV related to the OH species or excess oxygen was observed in some cases [55]. However, it is almost unobservable in this case, implying that the precursors are completely reacted on the substrate surface, otherwise the OH-related species would remain in the films. In addition, the area ratio of O_D_/(O_D_ + O_L_) is calculated to indicate the concentration of oxygen vacancy defects. The area ratio decreases from 15.6% to 11.2% as the annealing temperature increases from 300 to 700 °C, which also indicates the suppression of oxygen vacancy defects. In addition, the increase of film thickness, interplanar distance of (100) planes and refractive index serve as the secondary evidence since they can be possibly induced by the suppression of oxygen vacancy defects. The oxide layer may increase as more oxygen atoms are incorporated in the film, thus increasing the film thickness. The lattice contraction may restore as the oxygen vacancy defects are repaired, leading to the increase of interplanar distance. The film density is also expected to increase as the vacancy defects are suppressed, leading to the increase of refractive index. However, the area ratio increases to 11.8% when the annealing temperature further increases to 800 °C due to the out-diffusion of the oxygen atoms.

**Figure 6:**
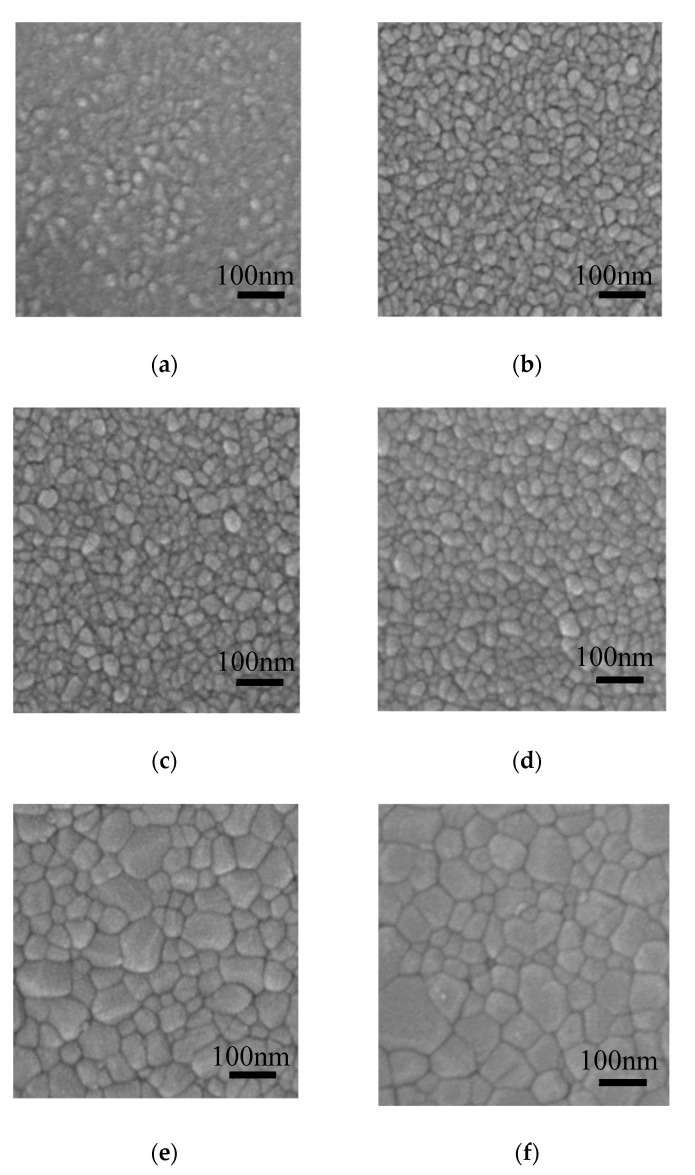
Field-emission scanning electron microscope (FESEM) images of sALD-ZnO films annealed at (**a**) 300 °C, (**b**) 400 °C, (**c**) 500 °C, (**d**) 600 °C, (**e**) 700 °C, and (**f**) 800 °C.

Figure 6 shows the FESEM images of the ZnO films deposited on silicon wafer and annealed at 300–800 °C. The film surface seems to be consisted of rice-like grains and amorphous phase when annealed at 300 °C. The grain size becomes larger accompanied by the transformation of amorphous phase to the crystalline phase as the annealing temperature increases, resulting in more compact film surface at high annealing temperature. The average lateral size of the grains was evaluated by the following method: firstly, a diagonal line was draw in the SEM images; then the number of grains under the diagonal line was counted out; finally, the average grain size was obtained by dividing the length of the diagonal line by the number of grains under it. The average lateral sizes of the grains were 21.0, 24.1, 30.2, 32.2, 45.3, 58 nm for the ZnO films annealed at 300, 400, 500, 600, 700 and 800 °C, respectively. The grain sizes were similar to the crystallite size estimated from Scherrer’s equation for the samples annealed at 300–600 °C. However, they were significantly larger than the crystallite size for the samples annealed at higher temperature of 700–800 °C. In addition, the grain shape changed from rice-like (typical shape for ZnO crystallite) at 300–600 °C to polygonal at 700–800 °C [31]. Therefore, we infer that the crystallites coalesce to form larger grains at such a high annealing temperature.

**Figure 7:**
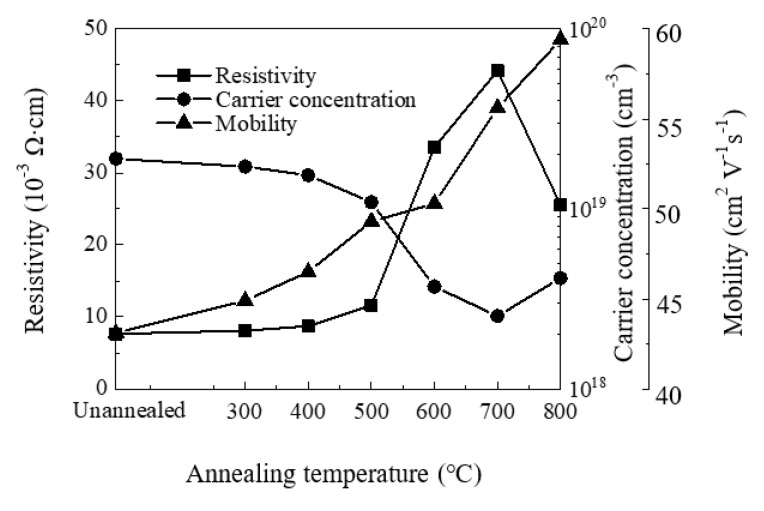
The variation of resistivity, carrier concentration and mobility of sALD-ZnO films with annealing temperatures.

Figure 7 shows the variation of resistivity, carrier concentration and mobility of the ZnO films with annealing temperature. We observe *n*-type conductivity for all films. The carrier concentration decreases with increasing annealing temperature at 300–700 °C, then slightly increases at 800 °C. Similar trends have been observed by other groups [57,58,59]. The decrease of carrier concentration should be ascribed to the suppression of oxygen vacancy defects since they have been identified as one of the origins of carrier concentration. In contrast, the increase of carrier concentration may be related to zinc or oxygen out-diffusion at 800 °C. The mobility increases with annealing temperature owing to the increase of crystallinity and crystallite size. The resistivity of the film increases with annealing temperature at 300–700 °C, then decreases at 800 °C. The variation of film resistivity should be ascribed primarily to the variation of carrier concentration since they exhibit close inverse relation. The decrease of (0 0 2) peak with increasing annealing temperature may also play a part in the increase of film resistivity. The resistivity decreases at 800 °C as the carrier concentration increases, which should be attributed to the oxygen out-diffusion at such a high temperature.

## 4. Conclusions

In this study, a 100-nm-thick ZnO film was prepared by a spatial ALD process with high deposition rate, which is promising for industrial applications. The oxygen vacancy defects in the ZnO film can be suppressed by annealing at 300–700 °C to improve the crystallinity and film properties, which might be helpful for some applications where oxygen vacancy defects are harmful and need to be suppressed. Annealing at a higher temperature of 800 °C might induce oxygen out-diffusion from the film and deteriorate the film properties. Therefore, it should be avoided in terms of suppressing the oxygen vacancy defects. In addition, the annealing process has little effect on the optical properties and a significant effect on the electrical properties of the ZnO film.

## Figures and Tables

**Table 1:** Parameters for the deposition and annealing process of spatial atomic layer deposition (sALD) ZnO film.

Parameter	Value
Substrate temperature (°C)	90
Moving speed of substrate holder (cm/s)	15
Gap between spray and substrate (mm)	0.3
Flow rate of H_2_O carry gas (sccm)	400
Flow rate of H_2_O dilute gas (sccm)	800
Flow rate of DEZ carry gas (sccm)	100
Flow rate of DEZ dilute gas (sccm)	3000
Concentration of DEZ precursor (cc/cm^3^)	40
Annealing ambient	air
Annealing temperature (°C)	300–800
